# Comparison of school based and supplemental vaccination strategies in the delivery of vaccines to 5-19 year olds in Africa - a systematic review

**DOI:** 10.12688/f1000research.12804.1

**Published:** 2017-10-13

**Authors:** Eposi C. Haddison, Leila H. Abdullahi, Rudzani Muloiwa, Gregory D. Hussey, Benjamin M. Kagina

**Affiliations:** 1 Vaccines for Africa Initiative (VACFA), University of Cape Town, Cape Town, South Africa; 2 School of Public Health and Family Medicine, University of Cape Town, Cape Town, South Africa; 3 Division of Medical Microbiology & Institute of Infectious Disease and Molecular Medicine, University of Cape Town, Cape Town, South Africa; 4 Department of Paediatrics & Child Health, Groote Schuur Hospital, University of Cape Town, Cape Town, South Africa

**Keywords:** Africa, systematic review, school based vaccination, supplemental immunisation activities, adolescents, school age children, routine immunisation

## Abstract

Background: Some vaccine preventable diseases (VPDs) still remain a public health burden in many African countries. The occurrence of VPDs in all age groups has led to the realization of the need to extend routine immunisation services to school age children, adolescents and adults. Supplemental immunisation activities (SIAs) and school based vaccinations (SBVs) are common strategies used to complement the expanded programme on immunisation (EPI). This review aimed to assess the effectiveness of SIAs compared to SBVs in the administration of vaccines to 5-19 year olds in Africa.

Methods: Systematic review methods were used to address our study aim. Several electronic databases were searched up to March 30, 2017 for primary studies investigating the delivery of vaccines via SIAs or SBVs to 5-19 year olds. This search was complemented by browsing reference lists of potential studies obtained from search outputs. Outcomes considered for inclusion were: vaccination coverage, costs of the strategy or its effect on routine immunisation services.

Results: Out of the 4938 studies identified, 31 studies met the review inclusion criteria. Both SIAs and SBVs showed high vaccination coverage. However, the SIAs reported higher coverage than SBVs: 91% (95% CI: 84%, 98%) versus 75% (95% CI: 67%, 83%). In most settings, SBVs were reported to be more expensive than SIAs. The SIAs were found to negatively affect routine immunisation services.

Conclusions: Both SIAs and SBVs are routinely used to complement the EPI in the delivery of vaccines in Africa. In settings where school enrolment is suboptimal, as is the case in many African countries, our results show SIAs may be more effective in reaching school age children and adolescents than SBVs. Our results re-iterate the importance of evaluating systematic evidence to best inform African authorities on the optimal vaccine delivery strategies targeting school age children and adolescents.

## Introduction

The Expanded Programme on Immunisation (EPI) was founded in 1974 to provide immunisation services to children, both nationally and globally ^1^. The EPI has proven to be a cost effective public health strategy, with reports suggesting that because of the programme, millions of infants’ lives globally have been saved against vaccine preventable diseases (VPDs) ^1^. Despite the widespread implementation of EPI, some VPDs still remain a public health burden in most of the African countries ^2, 3^. Suboptimal vaccination coverage rates in children targeted by the EPI and the inability to expand vaccination services to populations not targeted by the routine immunisation are some of the likely contributors to the high prevalence of VPDs in Africa ^4, 5^.

Routinely, school aged children and adolescents are not the primary target of EPI and as a result, an immunisation gap among these age groups has been observed in many settings ^6^. To address the immunisation gap, the WHO has recommended the inclusion of several vaccines targeting school aged children and adolescents in national immunisation programmes (NIPs). The WHO recommended vaccines for older children include those against the following pathogens: human papillomavirus (HPV), diphtheria, tetanus, pertussis, measles, rubella, hepatitis B and meningococcus ^6, 7^. A majority of the High Income Countries (HICs) have implemented the WHO recommendations of vaccinating the older children, but this is not the case with the Low and Middle Income Countries (LMICs) ^8– 10^.

Several reasons justify the inclusion of school aged children and adolescents into NIPs. First, infants who miss routine vaccinations remain susceptible to VPDs later in life ^6, 11^. Second, immunity acquired through infant immunisation for some VPDs like tetanus and pertussis wanes over time, thus requiring booster doses later in life ^12^. Third, there are epidemiological changes of some VPDs like rubella where more infection rates have shifted from infancy to adolescence, thus requiring a review of immunisation policies ^13^. Lastly, new vaccines under development such as against HIV and tuberculosis (TB) are likely to target older children and adolescents. In the absence of structured vaccine delivery programs for school age children and adolescents, many settings use school based vaccinations (SBVs) and supplementary immunisation activities (SIAs).

Supplementary immunisation activities, also known as mass vaccination campaigns refer to an immunisation strategy where a large number of people are vaccinated within a defined geographical area and period, regardless of previous vaccination status ^14^. The success of SIAs in disease outbreak control as well as in the eradication of smallpox is well documented ^14– 17^. However, there are reports suggesting negative effects of SIAs on the routine health services, including EPI ^18, 19^.

School based vaccinations (SBVs) target school going children on school premises and within school hours. The SBVs delivery strategy is newer to the EPI compared to SIAs ^20^, particularly in Africa. Currently, and in Africa, the main vaccine administered through SBVs is against HPV. Advantages of SBVs include high vaccination coverage and the possibility to extend other health services offered to school age children ^21– 23^. However, in Africa, there are millions of children not attending school ^24^ and are missed by SBVs strategy.

Our study aimed to compare the effectiveness of using SIAs or SBVs to deliver vaccines to 5–19 year olds in Africa.

## Methods

This review adhered to the Preferred Reporting Items for Systematic Reviews and Meta-Analyses (PRISMA) Checklist ( Supplementary File 1). The protocol was registered with PROSPERO (CRD42017057475) ^25^.

### Search strategy

A search was carried out to identify all relevant studies. Both published and unpublished literatures were searched up to March 30, 2017. No restriction was placed on the publication language. The following electronic databases were searched using both medical subject headings (MeSH) and free text terms relating to vaccination, children, adolescents and Africa ( Supplementary Table 1): PubMed, Africa Wide, Cochrane Central Register of Controlled trials (CENTRAL), Cumulative Index to Nursing and Allied Health Literature (CINAHL), World Health Organization Library Information System (WHOLIS), Web of Science, PDQ (Pretty Darn Quick)-Evidence and Scopus. The following grey literature databases were searched for reports, non-peer reviewed and non-indexed papers: WHO, The Global Vaccine Alliance (GAVI) and UNICEF. The reference lists of the included publications were evaluated to identify other potential studies.

### Study selection

The following criteria was used to select primary studies for inclusion:

(1) the study was either a randomised controlled trial (RCTs), non-RCT, cluster-RCT, interrupted time series, controlled before-and-after, cohort, cross-sectional or case-control; 

(2) participants were school aged children or adolescents (5–19 years) living in Africa;

(3) SIAs or SBVs were the vaccination strategies under investigation;

(4) vaccination coverage, cost of vaccine delivery or effects of either strategy (SIAs or SBVs) on routine health services including EPI were reported as any of the outcomes. Retrieved articles were independently screened by two reviewers (HEC and LA). Where study eligibility was unclear, a review was carried out by a third independent study team member (BK).

### Data extraction

HEC and LA independently reviewed each included study and extracted data using a piloted data extraction form. Where discrepancies arose HEC, LA and BK reached a consensus by discussion. Corresponding authors were contacted for any missing data needed during the extraction process.

### Quality assessment of included studies

Experimental studies were assessed using the Cochrane Collaboration’s tool for assessing risk of bias ^26^, while the Hoy *et al.,* modified tool was used for cross-sectional studies ^27^. Using the Cochrane checklist, studies were scored as ‘High risk’ or ‘Low risk’. Using the Hoy *et al.,* checklist, cross-sectional studies were scored as High risk’ or ‘Moderate risk’ or ‘Low risk’ of bias. Studies with high risk of bias were rated as poor quality while studies with moderate and low risk of bias were rated as moderate and high quality respectively. Where discrepancies in quality assessment occurred, HEC and LA discussed to arrive at a consensus. The quality of studies reporting the cost effectiveness was not assessed since our main interest was only the delivery costs.

### Data synthesis and analyses

Data was analysed using Stata v. 14.0. Results from the studies reporting vaccination coverage were expressed as percentages. Reported costs of the delivery strategies were standardised to United States Dollars (USD) if reported in a different currency. The costs of the strategies (SBVs or SIAs) and effects of the strategy on routine vaccination were presented in a narrative form.

A meta-analysis for vaccination coverage using a random effects model with inverse variance proportion was carried out. Pooled statistics for vaccination coverage were expressed as proportions with 95% confidence interval (95% CI). Subgroup analyses were carried to evaluate vaccination coverage per strategy, stratified by study settings or type of vaccine. Missing values were imputed in order to enable a complete case analysis. A sensitivity analysis was then carried out where imputations were done to assess if the results significantly differed due to the data imputations.

Visual inspection of the forest plots, the Chi-squared test for homogeneity with a significance level set at 10% and the I ^2^ statistic were used to assess and quantify any heterogeneity between study results. Heterogeneity was rated as ‘low’ for ≤ 49% and ‘moderate’ for 50–74% and ‘high’ for ≥ 75% using the I ^2 ^statistic.

## Results

### Literature search

Three thousand seven hundred and nineteen (3719) studies were identified through searching the electronic databases. A further 1461 were identified from grey literature. An additional five studies were identified from the reference lists of all the search outputs. After duplicates were removed, 4938 studies were left for screening. The titles and abstracts of the 4938 studies were screened and 4872 were not relevant and therefore excluded. The full text of the remaining 65 were retrieved and assessed for eligibility. From the 65 studies, 31 met our inclusion criteria ( Supplementary File 2).

### Characteristics of included studies

**Figure 1.**
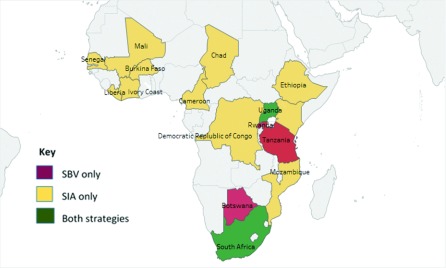
Countries and vaccine delivery strategies represented by the 31 included studies.

A total of 31 studies were included in this review. There were 20 cross-sectional studies ^23, 28– 46^, eight economic evaluation studies ^47– 54^, one cluster-randomised trial ^55^, one epidemiological report ^56^ and one interrupted time series ^57^. The included studies were published between 1993 and 2016; only three studies were published before 2000 ^45, 46, 56^. A total of 17 African countries ( Figure 1) and five different vaccines were represented from the included studies. Except for four of the included studies that were written in French ^44, 46, 50, 53^, the rest were in English. One of the study team members (HEC) is French literate and translated the four articles. In terms of vaccine delivery strategy, 20 and 11 studies assessed SIAs and SBVs respectively. Out of these 32 studies, 20 reported on vaccination coverage ( Table 1 and Table 2), nine on the cost of the vaccination strategy ( Table 3) and three on the effect on routine immunisation ( Table 4).

**Table 1.** Characteristics of the included studies reporting vaccination coverage for SIAs.

First author and year of publication	Setting	Vaccine	Targeted population | Vaccinated population	Age group of interest	Coverage
Ouatara *et al.*, 2013	Urban/rural	Meningitis (PsA-TT)	817 | 782	6–15	95.6%
Meyer *et al.*, 2015	Urban/rural	Meningitis (PsA-TT)	10001 | 9741	6–15	97.4%
Tall *et al.,* 2015	Urban	Meningitis (PsA-TT)	232 | 210	5–19	90.5%
Luquero *et al*., 2011	Urban	Measles	-	5–15	95%
Spiegel *et al.*, 1993	Urban	Meningitis (bivalent A C)	850 | 833	5–19	98%
Gil Cuesta *et al.,* 2015	Urban	Measles	-	5–15	87.4%
Ohuma *et al.*, 2009	Rural	Measles	378 | 334	5–15	88.3%
Huhn *et al.*, 2005	Displaced	Yellow Fever (17D)	25230 | 12238	5–14	48.5%
Cavailler *et al.*, 2006	Urban	Cholera (rBS-WC)	-	5–14	62.1%
Bagonza *et al.*, 2013	Rural	Yellow Fever	201 | 197	5–15	98%
CDC, 1999	Urban/rural	Measles	4045498 | 3495415	5–14	86%
Verguet *et al.*, 2013	Urban/rural	Measles	10383500 | 7579955	5–14	73%

**Table 2.** Characteristics of included studies reporting vaccination coverage for SBV.

First author and year of publication	Setting	Vaccine	Selection criteria	Targeted population | Vaccinated population	Age range	Coverage
Raesima *et al.*, 2015	Urban	HPV	Grade/Age	2488 | 1967	9–14+	79%
Binagwaho *et al.*, 2012	Urban/rural	HPV	Grade	94141 | 88927	12	94.4%
Moodley *et al.*, 2013	Rural	HPV	Grade/Age	963 | 938	9–14	97.4%
Snyman *et al*., 2015	Rural	HPV	Grade/Age	965 | 495	9–14	51.2%
Botha *et al*., 2015	Urban/rural	HPV	Grade	3465 | 1859	9–12	53.7%
Watson-Jones *et al.,* 2012	Urban/rural	HPV	Grade/Age	5532 | 4211	12–13	76.1%
La Montagne *et al.,* 2011	Rural	HPV	Grade	2008: 3459 | 3131 2009: 2835 | 2512	-	90.5% 88.6%
Katagwa *et al*., 2014	Rural	HPV	Age	415 | 176	9–19	42.4%

**Table 3.** Characteristics of included studies reporting the cost of the vaccination strategy.

First author and year of publication	Setting	Targeted age group (Years)	Vaccine	Vaccinated population	Cost of strategy USD	Cost per fully immunised person	Major sources of expenditure (%)
**School based vaccinations**							
Quentin *et al*., 2012	Urban/Rural	12–13	HPV	4,211	349,367	66–107	Salaries (42) Procurement (34)
Levin *et al.*, 2013	Rural	Grade 5	HPV	3,038	306,463	9.5	Salaries (40) Start-up costs (27)
**Supplemental immunisation activities**							
Verguet *et al*., 2013	Urban/Rural	5 – 14	Measles/ Polio	12,649,448	36,859,000	2.9	Personnel (58.3) Vaccines (30.6)
Zengbé-Acray *et al*., 2009	Urban	> 6 months	Yellow fever	2,610,994	2,382,582	0.9	Vaccines and consumables (80.6)
Legros *et al.,* 1999	Displaced	≥ 1	Cholera	27,607	14,655	0.53	Transport of vaccines (61.8) Consumables (21.8)
Wallace *et al*., 2014	Rural	6 months– 15	Measles	457,035	380,052	72.29	Vaccines and consumables (67) Salaries (23)
da Silva *et al*., 2003	Urban/rural	1–25	Yellow fever/ Meningitis A/C	85,925	62,055.44	72.2	Vaccines and consumables (86)
Uzicanin *et al.*, 2004	Urban/rural	9 months – 14	Measles	- -	Western Cape: 927,287 Mpumalanga: 781,858	0.96 0.87	Vaccine administration (73%)
Schaetti *et al*., 2012	Urban/rural	≥ 2	Cholera	23,921	760,000	30	Vaccines (67.1) Salaries of international staff (14.4)

**Table 4.** Characteristics of included studies reporting effect on routine immunisation.

First author and year of publication	Country	Setting	Vaccine	Duration of strategy	Effect
**Supplemental immunisation activities**					
Mounier-Jack *et al*., 2014	Mali	Urban/rural	Meningitis (PsA-TT)	10 days	Negative effect. Fewer children vaccinated through routine immunisation during vaccination campaign than expected.
Verguet *et al*., 2013	South Africa	Urban/rural	Measles	3 weeks	Negative effect. The use of child health services decreased during the vaccination campaign
**School based vaccinations**					
Torres-Rueda *et al*., 2016	Rwanda	Urban/rural	HPV	2 days	No or minimal effect. Routine immunisation continued during the vaccination campaign with the same demand for services.

### Risk of bias and quality assessment

**Figure 2.**
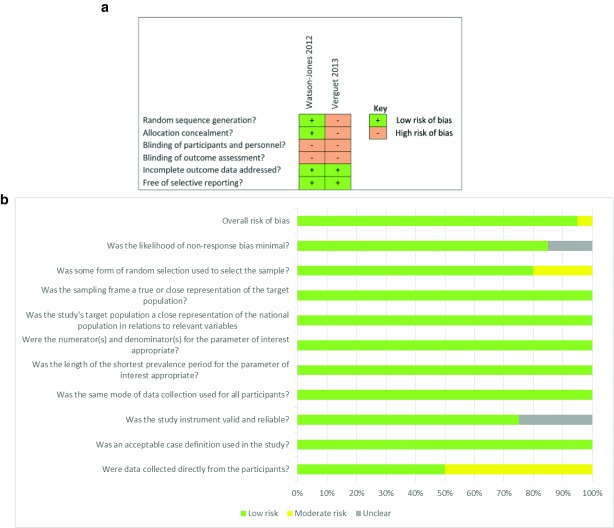
**a**. Risk of bias for experimental studies. **b**. Risk of bias for cross-sectional studies.

Using the Hoy *et al.,* modified tool ^27^, a 10 item scale was used to assess the internal and external validity of the 20 cross-sectional studies. Ninety-five percent (19) of the studies were of high quality (low risk of bias) meaning further research is unlikely to change our confidence in the estimate of the study outcomes. Five percent (1) of the studies were of moderate quality (moderate risk of bias) meaning further research is likely to have an impact on our confidence in the estimate of the outcomes ( Figure 2b). For the internal validity, the included studies defined which participants were considered to have been vaccinated (by self-report or vaccination card) and used the same data collection tool for all the participants. However, five studies did not mention if the tool used was standardised ^29, 34, 42, 45, 46^. Ten studies collected information from proxies (parents or guardians of vaccinated children) ^29– 32, 34, 36, 43– 46^. All the studies calculated vaccination coverage as the ‘number vaccinated divided by the number of the targeted population’. For the external validity, all the studies had representative samples in terms of age and sex. Random sampling was used in all except four studies ^33, 35, 37, 39^. Similarly, majority of the studies had a low non-response rate except three studies ^33, 40, 46^.

The Cochrane checklist was used to assess the clustered-randomised trial ^55^ and interrupted time series ^57^ study ( Figure 2a). 

### Vaccination coverage

**SIAs:** Twelve studies reported vaccination coverage for SIAs. Three studies reported coverage data on meningococcal serogroup A (PsA-TT) vaccine ^29, 30, 44^, five on measles vaccine ^31, 32, 34, 56, 57^ and two on yellow fever vaccine ^36, 43^. Each of the remaining two studies reported on one vaccine; cholera ^38^ and meningococcal bivalent polysaccharide A/C vaccine ^46^. Vaccination coverage for SIAs ranged from 48.5% to 98% ( Table 1).

**Figure 3.**
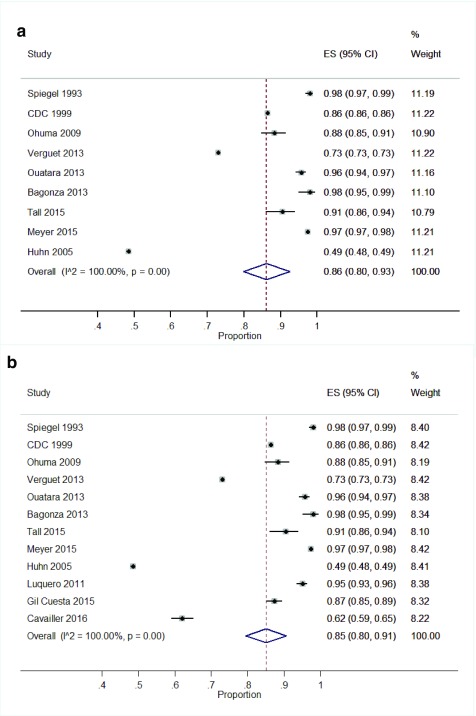
**a**. Forest plot showing vaccination coverage for SIAs. **b**. Forest plot showing vaccination coverage for SIAs with imputed data.

A meta-analysis of a pooled estimate showed high vaccination coverage for SIAs 86% (95% CI: 80%, 93%) ( Figure 3a). Three studies were excluded from the pooled meta-analysis due to missing sample sizes ^31, 32, 38^. However, after imputation of the sample sizes based on vaccination coverage, no major difference was seen in the pooled coverage ( Figure 3b).

**Figure 4.**
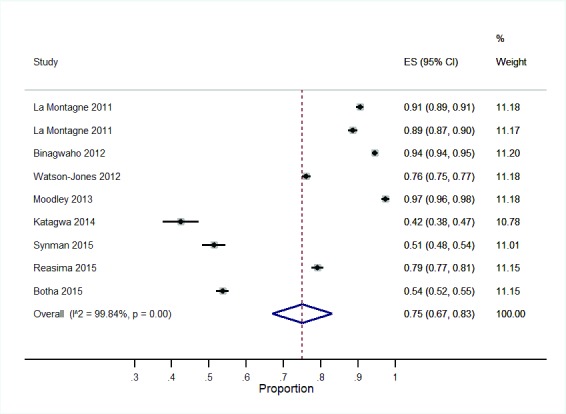
Forest plot showing vaccination coverage for SBV.

**SBVs:** Eight studies reported vaccination coverage for SBVs ( Table 2). All the SBVs reported coverage of the HPV vaccine among girls aged 9–19 years. Four HPV vaccine studies reported a combination of a grade and age based approach for identifying the target girls for the vaccination ^28, 33, 35, 55^ while three studies reported a grade based only approach ^23, 40, 41^. The remaining study identified girls for HPV vaccination by age ^42^, ( Table 2). Vaccination coverage of completed doses among the targeted population ranged from 42.4 – 97.4%. A meta-analysis of SBVs showed a pooled vaccination coverage of 75% (95% CI: 67, 83) ( Figure 4).

**Figure 5.**
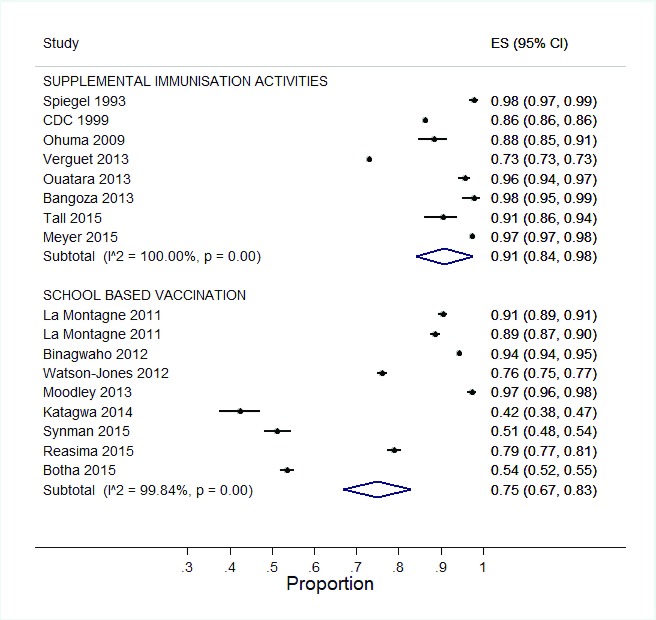
Comparison of vaccination coverage per strategy.

**Comparison of vaccination coverage:** To compare the two vaccine delivery strategies, pooled estimates for SIAs and SBVs were evaluated. For this comparison and subsequent analyses, Huhn *et al*., study ^36^ was not included since its setting (displaced) is not similar to the other studies reporting SIAs. The SIAs showed a higher vaccination coverage than and SBVs ( Figure 5).

### Subgroup analyses for vaccination coverage

Subgroup analyses were carried out to evaluate if vaccination coverage varied by the study setting or by vaccine type (for SIAs).

**Figure 6.**
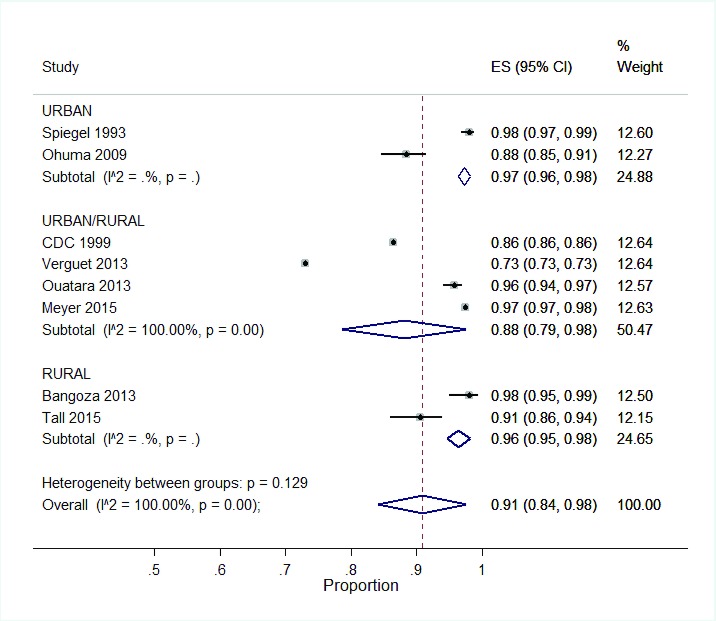
Subgroup analysis of SIA coverage per setting.

**Study setting:** The settings evaluated were: urban only, rural and urban mixed, or rural only. For SIAs, vaccination coverage did not vary irrespective of the three study settings ( Figure 6). However, vaccination coverage was highest in urban areas (97%, 95% CI: 96, 98) and lowest in a mixed setting (88%, 95% CI: 79, 98).

Similarly for SBVs, there was no variation in vaccination coverage across the three settings. Vaccination coverage in urban, rural and mixed settings were as follows: 79% (95% CI: 77, 81), 74% (95% CI: 63, 85) and 75% (95% CI: 53, 97) respectively.

**Figure 7.**
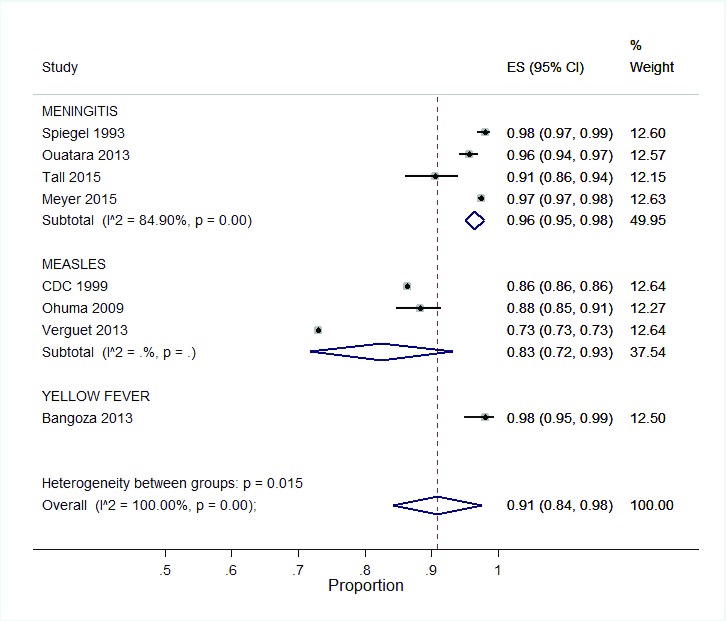
Subgroup analysis of SIA coverage per vaccine.

**Vaccine type:** There were variations in vaccine coverage dependent on the type of vaccine delivered via SIAs ( Figure 7). Of all the vaccinations, measles SIAs reported the lowest coverage 83% (95% CI: 72, 93). Highest vaccination coverage were reported for SIAs against meningitis and yellow fever at 96%, (95% CI: 94, 98) and 98% (95% CI: 95, 99) respectively.

### Cost of vaccine delivery strategy

**SIAs:** Seven studies reported the costs of conducting SIAs ( Table 3). The costs represent monies spent to deliver the vaccines to the total target population during the SIAs. The available data on costs of the SIAs was not age specific and therefore, the costs for 5–19 year olds only could not be calculated. The SIAs targeted children aged 6 months and older age groups with the number of vaccinated people across all studies ranging from 23921 to 12,649,448.

Majority of the total cost in SIAs was used to procure vaccines and consumables. The highest proportion (86%) for vaccines and consumables costs’ was reported by da Silva *et al*., for the combined yellow fever and meningitis A/C campaign in Senegal ^53^. Costs for salaries of health staff and international supervisors were another high expense during SIAs. Other reported minor costs were: training of personnel, social mobilisation, transport, maintenance of equipments, cold chain and management of adverse events following immunisation (AEFI).

**SBVs:** Two studies reported the cost of HPV vaccination in Tanzania and Uganda ^47, 48^ ( Table 3).In Tanzania, 4211 girls were vaccinated with three doses of HPV vaccine either based on age or class. The total economic costs for the SBVs were 349,400 USD. The total cost per fully immunised girl in urban areas were 66 USD and 100 USD for class-based and age-based approach respectively; and in rural areas, 78 USD and 107 USD for class-based and age-based approach respectively.. Administration and supervision (salaries) of the project and procurement of vaccines accounted for the major expenses. The reported minor expenses in the study included training, cold chain, waste management and social mobilisation. The observed difference between costs per fully immunised girl for class-based and aged-based selection may be due to the fact that more time and logistics were needed to select girls based on their age. In Uganda, 3038 girls in a rural setting were vaccinated with three doses of HPV vaccine using a class based approach. The total economic cost (cost of all resources used regardless of who paid) was 30,646 USD excluding the cost of the vaccine. The total economic cost per fully immunised girl was 9.5 USD. Salaries of staff accounted for the greatest part of the cost followed by micro-planning, staff training, community mobilisation (start-up costs). Other expenses included supplies, cold chain, vehicles and transportation.

### Effect of vaccination strategy on routine immunisation

**SIAs:** Two studies reported the effect of SIAs on routine immunisation and health services. Mounier-Jack *et al.,* reported on a 10 day meningitis A campaign in Mali, in 2010 ^37^ while Verguet *et al*., reported on a 3 weeks measles campaign in South Africa in the same year ^57^. Both SIAs reported a negative effect on routine immunisation ( Table 4). 

*Low attendance:* Both studies reported a decrease in the number of children attending the child clinics during the vaccination period. Mounier-Jack *et al.,* reported a 71–74% decrease in the number of children vaccinated during the campaign ^37^ while Verguet *et al.,* reported an 8% decrease in the number of children who were weighed in the postnatal clinic ^57^.

*Redeployment of health staff:* During the SIAs, staff in charge of routine immunisations were either deployed as supervisors for the campaign. This led to the closure of routine immunisation services in some districts during the campaign period.

*Cold chain management:* In Mali, routine vaccines were relocated and stored at the regional and district level so fridges could be made available to store the campaign vaccine ^37^.

**SBVs:** One study in Rwanda reported the effect of HPV vaccination on routine immunisation ^39^ ( Table 4). According to Torres-Rueda *et al.*, the vaccination activity which lasted 2 days had no effect on routine immunisation services ^39^. There was no change in the demand for routine immunisation and health services.

Characteristics of included studies reporting vaccination coverage Click here for additional data file. Copyright: © 2017 Haddison EC et al. 2017 Data associated with the article are available under the terms of the Creative Commons Zero "No rights reserved" data waiver (CC0 1.0 Public domain dedication).

## Discussion

Both SIAs and SBVs are supplementary EPI programs in many settings, including Africa. Our results show both strategies attain high coverage with SIAs showing greater vaccination coverage than SBVs. However, this coverage should be interpreted with caution since single dose vaccines were administered during the SIAs as opposed to two or three HPV doses during SBVs. Nonetheless, this review showed SIAs negatively affect the provision of routine health services, particularly the EPI. In settings like Africa where many resource challenges prevail, the simultaneous use of both SIAs and SBVs to reach school age and adolescents is questionable.

The high vaccination coverage achieved by SIAs reported in this review corroborates with the past successes of smallpox eradication, achieved by complementing EPI with SIAs ^58^. Interestingly, coverage of the SIAs was high irrespective of the vaccine and setting, and this attests to the robustness of the strategy. Despite being a new strategy in Africa and being used for the introduction of HPV vaccines, SBVs was able to achieve a high but variable coverage. Our findings are similar to those obtained in HICs where the SBVs strategy is more established and used for routine immunisation to this age group ^59^.

In terms of vaccine coverage, our review supports what is already known: both SIAs and SBVs are good options to complement the EPI. However, other factors such as the costs of the strategy, logistical requirements, the number of vaccine doses to be administered, school attendance and existing immunisation policies have to be taken into consideration when deciding which of the two strategies to use in any given setting. Local evidence should be used to evaluate which vaccine delivery strategy is more optimal to reach school age children and adolescents in Africa.

SBVs are likely to be more cost-effective than SIAs in countries with high school enrolment. Similarly, SBVs are likely to be optimal in countries with strong inter-ministerial collaboration. Collaboration between health and education sectors is crucial to ensure smooth implementation of SBV strategy ^60^. Conversely, SBVs are unlikely to be optimal in countries without sufficient financial commitment. School based vaccinations have been reported to be an expensive strategy which may be feasible on a small scale but not sustainable at a national level ^61^. Our findings support the reports that SBVs are an expensive strategy although this was based on limited data of newer and more expensive HPV vaccines.

Supplemental immunisation activities, could be the preferred strategy in countries where campaigns are regularly used to complement infant immunisation. The experience in conducting SIAs and community awareness of the strategy can be used to extend the vaccination services to school age children and adolescents. Additionally, the SIAs are able to reach non-school going children. A majority of African countries have millions of non-school going children ^24^ who will miss vaccines delivered by SBV programs. The negative impacts of SIAs on routine immunisation and health services due to the overlapping of resources (financial and human) is a key concern that should be minimized wherever SIAs are used.

### Strengths and limitations

We comprehensively and systematically searched for the relevant literature: both peer-reviewed and non-reviewed records were obtained. This study adhered to the PRISMA guidelines of conducting systematic reviews. Nonetheless, our review had several limitations. First, age specific coverage was not reported in some of the retrieved studies. Second, we observed a high heterogeneity during the meta-analysis. The heterogeneity was likely due to differences across studies of factors such as the age groups included, study settings and period. Third, the only vaccine administered via SBVs was the HPV vaccine, a new and more expensive vaccine. Lastly, few studies reported the effect of SIAs or SBVs on routine immunisation and health services and therefore, our findings may not accurately reflect the effects.

### Implication for policy and research

Local evidence is crucial in the review and development of immunisation policies. Both SIAs and SBVs are routinely used in Africa to vaccinate older children. In settings where school enrolment is low as is the case in many African countries, our results show SIAs may be more effective in reaching school age children and adolescents than SBVs. However, caution needs to be exercised to mitigate the negative effect of SIAs on the routine health services. In Africa, the SBV has mainly been tested in the delivery of two or three dose HPV vaccine to adolescent girls while SIAs have been used for diverse vaccines and on a larger scale. Further research is therefore needed to assess the sustainability of SBVs for nationwide delivery of vaccines to school age children and adolescent in resource constraint settings as is the case in Africa. In the event that SBVs are chosen as the main delivery strategy, complementary community activities can be set up to target out of school children. Our results re-iterate the importance of systematic evidence to best inform African authorities on the optimal delivery strategies of vaccines targeted at school age children and adolescents for immunisation.

## Data availability

The data referenced by this article are under copyright with the following copyright statement: Copyright: © 2017 Haddison EC et al.

Data associated with the article are available under the terms of the Creative Commons Zero "No rights reserved" data waiver (CC0 1.0 Public domain dedication). 

Dataset 1: Characteristics of included studies reporting vaccination coverage. DOI, 10.5256/f1000research.12804.d180616 ^62^.

## Supplementary material

**Supplementary File 1: PRISMA Checklist.**

**Supplementary File 2: PRISMA flowchart, showing the number of records identified, included and excluded.**

**Supplementary Table 1: Search strategy.**
