# Supplementary figures and images for: Comparison of school based and supplemental vaccination strategies in the delivery of vaccines to 5-19 year olds in Africa - a systematic review
Source: F1000Res. 2017 Oct 13;6:1833. [Version 1] doi: 10.12688/f1000research.12804.1 (PMC5765397; doi:10.12688/f1000research.12804.1)

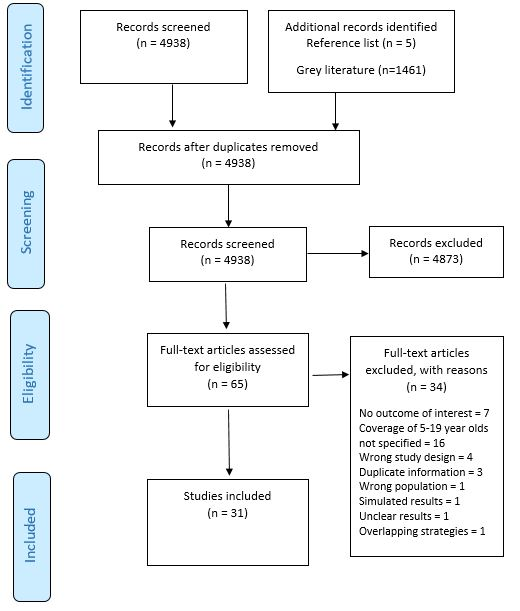

Supplement: Supplementary file 3 [file f1000research-6-13874-s0002.tgz › 49a144c5-46f6-4e04-a437-7c9438d209fe.tif]
