# Supplementary material for: Comparison of school based and supplemental vaccination strategies in the delivery of vaccines to 5-19 year olds in Africa - a systematic review
Source: F1000Res. 2017 Oct 13;6:1833. [Version 1] doi: 10.12688/f1000research.12804.1 (PMC5765397; doi:10.12688/f1000research.12804.1)
Supplement: Supplementary file 4 [file f1000research-6-13874-s0003.tgz › 4d1f55a3-75b5-40b2-a2c0-4d523a5aa0e7.pdf]

S1 Table. SEARCH STRATEGY

	**Search terms**
#4	#1 AND #2 AND #3
#3	Angola OR Republic of Angola OR Algeria OR The People's Democratic Republic of Algeria OR Botswana OR Benin OR Dahomey OR Republic of Benin OR Burkina Faso OR Burkina OR Republic of Upper Volta OR Burundi OR Republic of Burundi OR Central African Republic OR Chad OR Republic of Chad OR Cameroon OR Republic of Cameroon OR Republic of Cameroun OR Cote D'ivoire OR Ivory Coast OR Republic of Cote D'ivoire OR Jamahiriya OR Djibouti OR Republic of Djibouti OR Arab Republic of Egypt OR Egypt OR Democratic Republic of the Congo OR DR Congo OR Congo-Kinshasa OR DRC OR Zaire OR Eritrea OR State of Eritrea OR Ethiopia OR Federal Democratic Republic of Ethiopia OR The Gambia OR Republic of the Gambia OR Ghana OR Republic of Ghana OR Gabon OR Gabonese Republic OR Guinea OR Republic of Guinea OR Guinea-Conakry OR Guinea-Bissau OR Republic of Guinea-Bissau OR Kenya OR Republic of Kenya OR Liberia OR Republic of Liberia OR Madagascar OR Republic of Madagascar OR Malawi OR Republic of Malawi OR The Warm Heart of Africa OR Mali OR Republic of Mali OR Mozambique OR Republic of Mozambique OR Libya OR State of Libya OR South Africa OR Tunisia OR Namibia OR Lesotho OR Kingdom of Lesotho OR Mauritania OR Mauritius OR Mayotte OR Morocco OR Kingdom of Morocco OR Nigeria OR Federal Republic of Nigeria OR Sao Tome and Principe OR Democratic Republic of Sao Tome and Principe OR Senegal OR Republic of Senegal OR Seychelles OR Sudan OR Republic of the Sudan OR North Sudan OR Swaziland OR Kingdom of Swaziland OR Ngwane OR Niger OR Republic of Niger OR Rwanda OR Republic of Rwanda OR Sierra Leone OR Republic of Sierra Leone OR Somalia OR Federal Republic of Somalia OR South Sudan OR Republic of South Sudan OR St Helena OR Tanzania OR United Republic of Tanzania OR Republic of Tanganyika and Zanzibar OR Togo OR Togolese Republic OR Uganda OR Republic of Uganda OR Western Sahara OR Zambia OR Republic of Zambia OR Zimbabwe OR Republic of Zimbabwe OR Rhodesia
# 2	Child [MeSH] OR Adolescent [MeSH] OR children OR teenagers
#1	Mass vaccination [MeSH] OR Mass immunization OR Mass vaccination OR vaccination campaigns OR immunization campaigns OR Supplemental immunisation activities OR school immunisation programs OR school vaccination strategies
