# Supplementary material for: Comparison of school based and supplemental vaccination strategies in the delivery of vaccines to 5-19 year olds in Africa - a systematic review
Source: F1000Res. 2017 Oct 13;6:1833. [Version 1] doi: 10.12688/f1000research.12804.1 (PMC5765397; doi:10.12688/f1000research.12804.1)
Supplement: Peer review history

# Peer review: Referee response for version 1

**DOI:** 10.5256/f1000research.13874.r28617

**Published:** 2018-01-16

**Authors:** Ifedayo Adetifa

**Affiliations:** 1 Epidemiology and Demography Department, KEMRI-Wellcome Trust Research Programme, Kilifi, Kenya

**General Comments**

 This is a useful article that reviews two essential approaches to the delivery of vaccines in older children/age groups in Africa

**Abstract**

1. Methods
  1. please list the electronic databases and add a line describing how quality assessment of retrieved articles.
2. Results
  1. Please include number of countries represented by the 32 studies included in the review
3. Conclusions
  1. The authors say, ‘Both SIAs and SBVs are routinely used to complement…’. This is incorrect based on the results reported in this review. SIAs are definitely more commonly/routinely used not SBVs.
  2. While the assertion in the 2 ^nd^ sentence is well known truth, authors have not provided any results in this review to support this.

**Introduction**

1. 2 ^nd^ paragraph, 1 ^st^ sentence- it is not entirely true that the immunisation gap results from the EPI not primarily targeting school aged children and adolescents. Gaps may result from poor coverage of childhood vaccinations, changing epidemiology, waning immunity requiring booster doses, etc.  I suggest this paragraph should be revised to highlight the need to find suitable platforms reach older children/adolescents with vaccines primarily targeted at them, to address immunisation gaps and to deliver boosters. This is mentioned in the 3 ^rd^ paragraph but can come earlier and merged with the 2 ^nd^ paragraph
2. 3 ^rd^ paragraph, 4 ^th^ sentence- The shift in susceptibility to older ages for rubella will be the consequence of introducing rubella vaccine with suboptimal coverage like is currently seen for measles in Africa and as already reported elsewhere. I suggest a revision here to make this clear given the setting for the review.
3. 5 ^th^ paragraph, 3 ^rd^ sentence- HPV is not the main vaccine administered through SBVs in Africa. The few pilot HPV SBV do not compare to the use of SBV for MR, YF and MeningA campaigns.
4. 5 ^th^ paragraph, 4th sentence- is high coverage with SBVs a given considering its dependence on school enrollment? In the few cases where high coverage is reported, is the denominator population all school-age children or just enrolled children?

 

**Methods**

1. Study selection, item 2- are school age children/adolescents the same as those enrolled? Should authors not consider differentiating between both or clarify that they mean the same thing for purposes of this review?
2. Study selection, item 4- it is unclear why outcomes around cost of vaccine delivery were not extended to cost effectiveness if the data was reported in retrieved articles. In my opinion, not doing this is a missed opportunity to increase the relevance of results here to policy makers.  
3. This will increase the relevance of results here to policy makers.
4. The last sentence under study selection should be started as a new paragraph
5. Quality assessment of included studies- as earlier I mentioned earlier, not including papers reporting cost effectiveness is a significant omission.
6. Data synthesis and analyses-
  1. It is my understanding that a complete case analyses what you do when you restrict analyses to complete datasets followed by imputation for missing values and analyses of datasets with imputed data for comparison? Please clarify
  2. It would be helpful to have some more description of how imputation was done and a summary of the extent of missingness so readers can assess the extent to which the results depend on imputed data.
  3. Why did authors use a 10% significance threshold?
  4. Are the categories of inconsistency arbitrary selections by authors or supported by a reference?

 

**Results**

1. Literature search- Most of this text can be left out or shortened since all of this information is displayed in SF2.
2. Characteristics of included study
  1. 1 ^st^ sentence is a repetition
  2. What is an epidemiological report?
  3. Last sentence page 4, are there 31 or 32 studies?
3. Risk of bias and quality assessment- a table showing the quality rating for each included study would be helpful. Perhaps this table can replace figures 2a and 2b (move to supplementary file) or quality is reported in an additional column in tables 1-4
4. Comparison of vaccination coverage
  1. I think it is more useful to compare the SIA vs SBV for the same vaccines. Coverage for vaccines requiring multiple doses in SIA or SBV should not be compared to those requiring a single dose. There are also other sociocultural and religious issues associated with the uptake of HPV vaccination in particular.
  2. Figure 5 may be more useful if pooled coverage estimates by vaccine are included
5. Subgroup analyses for vaccination coverage
  1. Please revise the 2 ^nd^ sentence “For SIAs, …”
6. Cost of vaccine delivery strategy
  1. SBVs-Some information about the unit cost of the vaccine would be useful. Also, whether the vaccine procurement was subsidized by GAVI or not.
  2. Summaries of the total economic cost per fully immunised child cannot be correct if economic costs do not include vaccine costs.
7. For the pooled coverage estimates, authors do not comment on heterogeneity/I-square results at all yet there is significant heterogeneity for many of the results. Is there an explanation for this?

 

**Discussion**

1. 1 ^st^ paragraph, 2 ^nd^ sentence- Pooled coverage estimates by vaccine is given in the results so beyond the caveat given for single vs. multiple dose vaccines, it would be useful to read authors’ opinion about the impact of heterogeneity studies in these estimates.
2. 2 ^nd^ paragraph-
  1. I am not sure I agree measles coverage by SIA should be described as high.
  2. As I alluded to earlier, HPV coverage in SBVs has to be interpreted with caution
3. 4 ^th^ paragraph- I am not convinced, at least by the results presented here that SBVs are likely to be more cost-effective even with high school enrolment. Authors need to provide more data and vaccine delivery costs are not the same as cost effectiveness of the strategy
4. Strength and limitations
  1. While it is good to see comments about heterogeneity, it is important to mention this in the results according to cut-offs given in the methods.
  2. While school enrolment in general is lower than it should be in Africa, one other limitation is data on school enrolment in the settings covered by included studies was not available
5. Implication for policy and research
  1. I think the conclusion here regarding enrolment and effectiveness of SIAs compared to SBV is speculative. There is simply not enough data in this review especially for SBV to support this.

I have read this submission. I believe that I have an appropriate level of expertise to confirm that it is of an acceptable scientific standard, however I have significant reservations, as outlined above.

# Peer review: Referee response for version 1

**DOI:** 10.5256/f1000research.13874.r28889

**Published:** 2018-01-10

**Authors:** Eddy Bresnitz

**Affiliations:** 1 Merck & Co., Inc., Kenilworth, NJ, USA

Well written report assessing the issue. Methods clearly described. Agree with general comments of the first reviewer. In addition to Dr. Burnett's editorial comments, I have several additional suggestions.

1. On page 4 in the Characteristics section, please clarify whether it's 31 or 32 studies reviewed.
2. In Tables 2 and 3, I suggest the column entitled "Targeted/Vaccinated" be inverted so that the numerator and denominator are in the appropriate relationship in the column.
3. I find that the provision of costs in Table 3 is not really helpful to policy makers. As written, the text just describes facts but does not provide an interpretation of the implications for other programs. Also, I'm not sure comparing SIV programs that use different vaccines, in different countries, is very helpful either. The cost per immunized person is very contextual.
4. Clearly, the SBV programs are limited to HPV whereas the SIV studies are a mélange of vaccines and approached. Perhaps there can be more discussion of how this (and the related social issues) could skew the results.

I have read this submission. I believe that I have an appropriate level of expertise to confirm that it is of an acceptable scientific standard.

# Peer review: Referee response for version 1

**DOI:** 10.5256/f1000research.13874.r28681

**Published:** 2017-12-11

**Authors:** Rosemary J. Burnett

**Affiliations:** 1 Department of Virology, Sefako Makgatho Health Sciences University, Pretoria, South Africa

**General comments:**

 This systematic review comparing SIAs to SBVs in Africa has been well designed and conducted, and the conclusions reached are well-founded. The manuscript can however be improved, with some minor editing. This includes deleting the repetition of phrases / words within a sentence / paragraph, as these make some sentences / paragraphs clumsy and increase the word count unnecessarily. Also, abbreviations must (a) only be introduced if they will be used again; (b) be introduced the first time the term is used and used only thereafter; and (c) never reintroduced. An edited version of the PDF has been sent to the supervisor to assist with improving and shortening the manuscript.

**Specific comments:**

 Introduction:

 Page 3, paragraph 3: The third point does not adequately explain the issue. The shift in age at infection has occurred because of the introduction of the MMR into the infant vaccination schedule followed by low coverage. Is this a problem in Africa? Surely not, since the MMR is not widely used. The third point here should thus be about vaccines needed before girls become sexually active, and should include both HPV and rubella.

 Page 3, paragraph 3, last sentence: School-based vaccinations are offered through structured vaccination delivery programs, but this sentence makes it sound as if they are not.

 Page 3, paragraph 5, 2 ^nd^ sentence: This suggests that in other regions, SBVs preceded SIAs, which is not the case.

 Page 3, paragraph 5, 3 ^rd^ sentence: Reference?

 Page 3, paragraph 6: The aim should be unpacked into objectives to clarify exactly what aspects of effectiveness are being measured.

 Methods:

 Page 3, last part of study selection: The last 2 sentences are not unique to (4), so should start on a new line.

 Page 4, end of paragraph 1: Because the objectives have not been clearly stated, the last sentence is not clear.

 Results:

 In general, what is clearly presented in the tables and figures should not be repeated in the text, as this makes the results section repetitive and too long. Also, be consistent in the way the 95% CI is reported. Conventionally it is xx-xx%, but whichever format is used, use it consistently throughout.

 Page 5, 1 ^st^ sentence under “risk of bias”: This sentence is part of the methods, not the results.

 Page 5, end of 3 ^rd^ sentence in paragraph under risk of bias: Figure 2b is mentioned in the text before Figure 2a.

 Page 5, Table 1 and Table 2: The country where the study was conducted should be included in these tables.

 Page 6, Table 3: The country where the study was conducted should be included in this table.

 Page 6, Table 3: Vergeut et al, column on major expenses: Does the word “Personnel” include more than salaries? If not, then this should be “salaries” to be consistent.

 Page 6, Table 3: Legros et al, column on major expenses: Transport is mentioned as a minor cost in the text, but vaccine transport is listed as a major cost here. Or is the transport being referred to in the text, transport of personnel? Clarity is needed.

 Page 6, Table 3: Uzicanin et al, column on major expenses: In the text, vaccine administration is summarised as salaries.

 Page 6, Table 3: Schaetti et al, column on major expenses: The salary detail for international staff is given here, which immediately raises a question about whether or not staff are local or international in the other studies.

 Page 7, Figures 2a and 2b: More details in the captions would add clarity, eg: number of studies, followed by reference numbers.

 Page 11 paragraph 2: Can be summarised as personnel salaries being the second major cost driver. Also, transport of vaccines is listed in table 3 in the column on major costs. Or is this transport of personnel?

 Page 11 paragraph 3: In the text, vaccine administration is summarised as salaries. See comment about this in table 3. Terms need to be used consistently for clarity.

 Page 11, 1 ^st^ paragraph under SIAs: Very clear in the table, thus unnecessary

 Page 11, 1 ^st^ sentence under “low attendance”: Again, this is clear both from the table and the text below this sentence.

 Page 11, 2 ^nd^ sentence under “redeployment of health staff”: Not stated in the table, and not clear if this was for both studies.

I have read this submission. I believe that I have an appropriate level of expertise to confirm that it is of an acceptable scientific standard.
